# A multi-centre student survey on weighing disciplines in medical curricula – a pilot study

**DOI:** 10.3205/zma001101

**Published:** 2017-05-15

**Authors:** Hormos Salimi Dafsari, Stefan Herzig, Jan Matthes

**Affiliations:** 1 Ludwig-Maximilians-University, Dr. von Hauner's Childrens' Hospital, München, Germany; 2 University of Cologne, Vice-Rector of Studies, Cologne, Germany; 3 University of Cologne, Centre of Pharmacology, Cologne, Germany

**Keywords:** curriculum, medical education, student participation, online survey

## Abstract

**Aim: **Initiated by students, this pilot study examines how obtaining medical students’ perspectives via a structured online survey may prove useful for curriculum deliberation.

**Methods:** In 2012, 747 students of 32 medical faculties in Germany assessed disciplines specified in the Medical Licensure Act (AÄpprO) thereby concerning the allocation of teaching time, perceived usefulness regarding preparation for state examination and medical practice, their interest and motivation for studying as well as consideration for future work.

**Results: **Internal medicine, surgery, paediatrics, gynaecology/obstetrics and general medicine rank amongst the upper third regarding allocation of teaching time and perceived usefulness for future medical practice. Concerning both preparation for state examination and medical practice internal medicine ranks second, while surgery only 22^nd^ and 28^th^ of 32, respectively. Some clinical-theoretical disciplines (e.g. pharmacology) are in the top ten regarding perceived preparation for state examination, too. Students who consider choosing internal medicine for future work rate associated disciplines significantly higher regarding usefulness for clinical practice (e.g. pharmacology) or motivation for studying (e.g. microbiology) than other students do.

**Conclusion:** A simple survey reveals interesting data on students’ perceptions and ideas of medical studies. Though the data are plausible, interpretations should be done with caution. Nonetheless, data like these should give rise to further questions and discussions, e.g. as part of curriculum deliberation.

## Zusammenfassung

**Zielsetzung: **Die studentisch initiierte Pilotstudie untersucht, inwiefern die Perspektive von Medizinstudierenden mittels einer strukturierten Befragung für die Curriculumsentwicklung nutzbar gemacht werden kann.

**Methodik:** 2012 machten 747 Medizinstudierende von 32 Fakultäten in ganz Deutschland online Angaben zu den von der Approbationsordnung seinerzeit für das klinische Studium vorgeschriebenen Fächern und Querschnittsbereichen u.a. in Bezug auf die Beimessung von Unterrichtszeit, Nützlichkeit für Staatsexamen und ärztliche Tätigkeit, Interesse und Lernmotivation sowie Erwägung für die spätere Tätigkeit.

**Ergebnisse:** Innere Medizin, Chirurgie, Kinderheilkunde, Frauenheilkunde-Geburtshilfe und Allgemeinmedizin liegen bei der Beimessung der Unterrichtszeit sowie der Bewertung der Nützlichkeit für den ärztlichen Alltag im oberen Drittel. Bezüglich der Vorbereitung auf Staatsexamen und ärztlichen Alltag liegt Innere Medizin jeweils auf Rang 2, Chirurgie nur auf 22 bzw. 28. Unter den Top 10 hinsichtlich der Vorbereitung auf das Staatsexamen finden sich auch klinisch-theoretische Fächer (z.B. Pharmakologie). Mit Bezug auf die eigene Perspektive fällt auf, dass z.B. Studierende, die sich Innere Medizin als Betätigungsfeld vorstellen können, assoziierte Fächer signifikant höher hinsichtlich der Nützlichkeit für die ärztliche Praxis (z.B. Pharmakologie) oder der eigenen Lernmotivation (z.B. Mikrobiologie) bewerteten als die übrigen Befragten.

**Schlussfolgerung:** Eine einfach gehaltene Befragung liefert interessante Ergebnisse zu studentischer Wahrnehmung und Vorstellung vom Medizinstudium. Die Daten sind plausibel, ihre Interpretation muss aber zurückhaltend erfolgen. Derlei Ergebnisse sollten Anlass zu gezielter Nachfrage und konstruktiver Diskussion z.B. im Rahmen von Curriculumsplanung und -ausgestaltung geben („curriculum deliberation“).

## Introduction

The German Medical Licensure Act (Ärztliche Approbationsordnung, ÄApprO) defines the disciplines in the clinical part of medical studies as well as their formats (§ 2) or the teaching time in total (§§ 2, 27, appendix 1 in § 2). However, content or teaching time of the certain disciplines are not defined [http://www.gesetze-im-internet.de/_appro_2002/index.html]. In 2015, the convention of medical faculties (Medizinischer Fakultätentag, MFT) adopted a nationwide catalogue of learning objectives (Nationaler Kompetenzbasierter Lernzielkatalog Medizin, NKLM) which describes the profile of medical graduates [1], [2]. Though the NKLM is intended as a guideline, eventually the particular faculties are responsible for the actual design of medical studies. Besides various recommendations on the conception of medical studies (e.g. [3]) mutual consent of disciplines’ representatives and curriculum directors is crucial here. Students get involved either within their faculties (e.g. committees, panels, task forces) or through transregional representation (e.g. the German Medical Students’ Association: Bundesvertretung der Medizinstudierenden in Deutschland e.V., bvmd). Since students experience medical education on a daily basis, it seems naturally to engage them into curriculum development [4]. Referring to Schwab’s concept of “curriculum deliberation”, Bordage and Harris recommended to involve those “who ‘live in’ or are strongly affected by the education programmes“ [5], [6]. It is desirable that these groups of persons get more involved, e.g. by surveys or the Delphi method. There are already examples for such approaches on a faculty level (e.g. [7]).

To examine the possibility of involving medical students in curricular development, we performed a nationwide survey on their evaluation of disciplines of the clinical part of medical studies as defined in the German Medical Licensure Act. Medical students were invited to prioritize to what extent disciplines should be allocated teaching time for theory or practice, and to evaluate each discipline’s usefulness for daily medical practice. Furthermore, they were asked to estimate their motivation for studying a particular discipline and to rate their perceived preparation for either state examination or daily medical routine, respectively. From the obtained data a kind of “desired curriculum” can be derived that may help to identify disciplines in putative need of adjustments. The comparison with actual curricula shows that assessments or “desires” of students to some extent already correspond with how faculties indeed conduct their curricula. However, we also observe some differences that should give rise to further discussion. Our study is a feasibility study regarding the questions how and to what extent a structured survey may contribute to the process of curriculum development. 

## Methods

### Survey 

This student-initiated study was designed according to the recommendations of the AAPOR report on online panels [8] and piloted amongst 20 members of the German Medical Students’ Association (bvmd e.V.). The survey consisted of five screens provided via the free of charge online platform SoSciSurvey [https://www.soscisurvey.de/index.php?page=home&l=eng]. Nationwide, medical students were invited by student representatives of all German medical faculties, the bvmd and via social media (Facebook, Twitter). A standardised invitation text gave information on study aims, the estimated time needed (about 10 minutes) and the anonymity of participation. The survey was available online for 41 days (11/20-12/31/2012). Participation was voluntary and without incentives. Via Likert scales, participants should evaluate the disciplines of the clinical part of medical studies defined by the German Medical Licensure Act. They were asked to assign the time for teaching theory or practice to each discipline (from 1=very little to 4=very much). Students were explicitly asked to prioritize in terms of ranking the different disciplines according to their individual views and desires and not to reflect the status quo. Two further items aimed at the perceived usefulness of a discipline and students’ motivation for studying it (from 1=not useful at all to 5=very useful, and from 1=not interesting to 5=very interesting, respectively). The participants were also asked which discipline they could imagine for their future work as a physician or researcher (multiple choices possible). Finally, they should state whether they felt well prepared for state examination and daily medical routine, respectively (from 1=disagree to 4=fully agree).

#### Participants

747 students (242 females) from 32 medical faculties in Germany participated. 19% were in the 1^st^ to 2^nd^, 64% in the 3^rd^ to 5^th^ years of their study, 17% in their (final) practical year. 65% of the participants came from six of the in total 36 medical faculties in Germany (Berlin n=103, Würzburg n=103, Cologne n=98, Hannover n=83, Aachen n=54 and Ulm n=47). Less than 40 students came from either of the other 30 faculties. Data obtained from study sites with ≥40 participants correlated well with the results of the other study sites (r>0,9: ”allocation of teaching time“, ”usefulness“ and ”motivation“; r>0,8: ”disciplines imagined for future work“; r>0,7: ”preparation for state examination“ and “preparation for daily medical routine”). Therefore, we judged the risk for a bias by sites with low participation or the domination of data by sites with high participation to be rather low.

#### Comparison with actual curricula

We compared the data from our survey with actually existing curricula from eleven German study sites (Berlin, Bonn, Cologne, Essen, Halle, Hannover, Kiel, Leipzig, Rostock, Ulm and Würzburg), running either reformed or conventional study programs or both. The information on the curricula were given by student representatives on site. 585 (77%) of the participants came from these 11 study sites.

#### Statistics

We conducted descriptive and explorative data analyses by using the “Statistical Package for the Social Sciences” (IBM SPSS Version 22). We used Pearson’s coefficient and Spearman’s rank correlation for analyses. For analyses of variance ANOVA or Kruskal-Wallis test were chosen as appropriate. P-values <0.05 were considered to indicate statistical significance.

## Results

### Allocation of teaching time – survey data versus actual curricula

**Figure 1:**
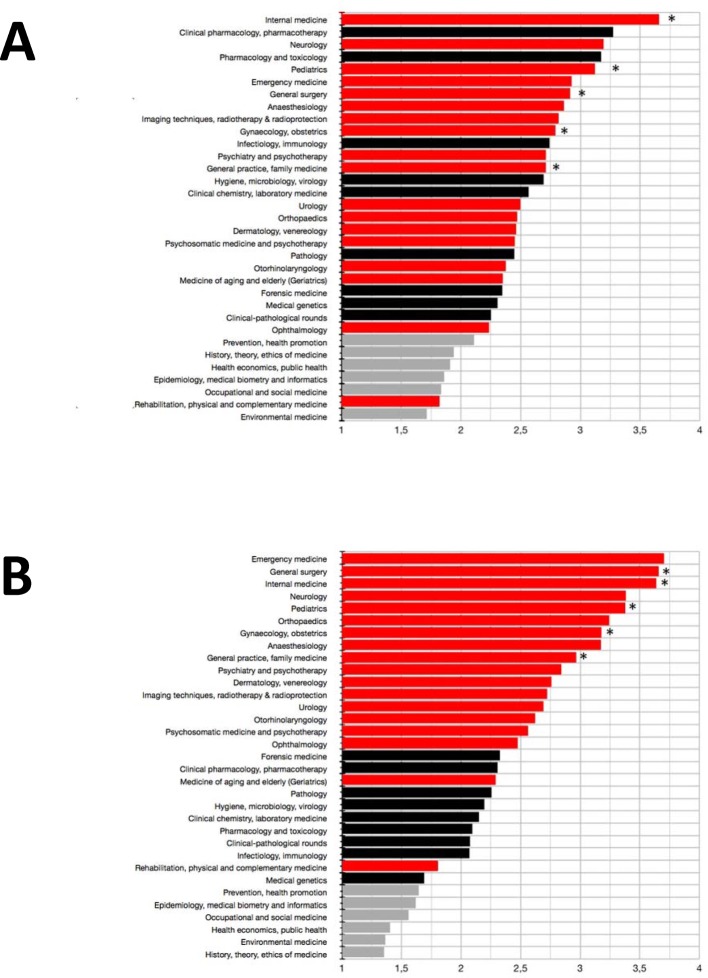
Relative allocation of teaching time for theory (A) and practice (B) regarding clinical-practical (red), clinical-theoretical (black) and socio-economic (grey) disciplines of the clinical part of medical studies. Rating was done via Likert scales (from 1=very little to 4=very much). Mean values are shown. *: disciplines with obligatory practical trainings according to the German Medical Licensure Act (ÄApprO).

**Figure 2:**
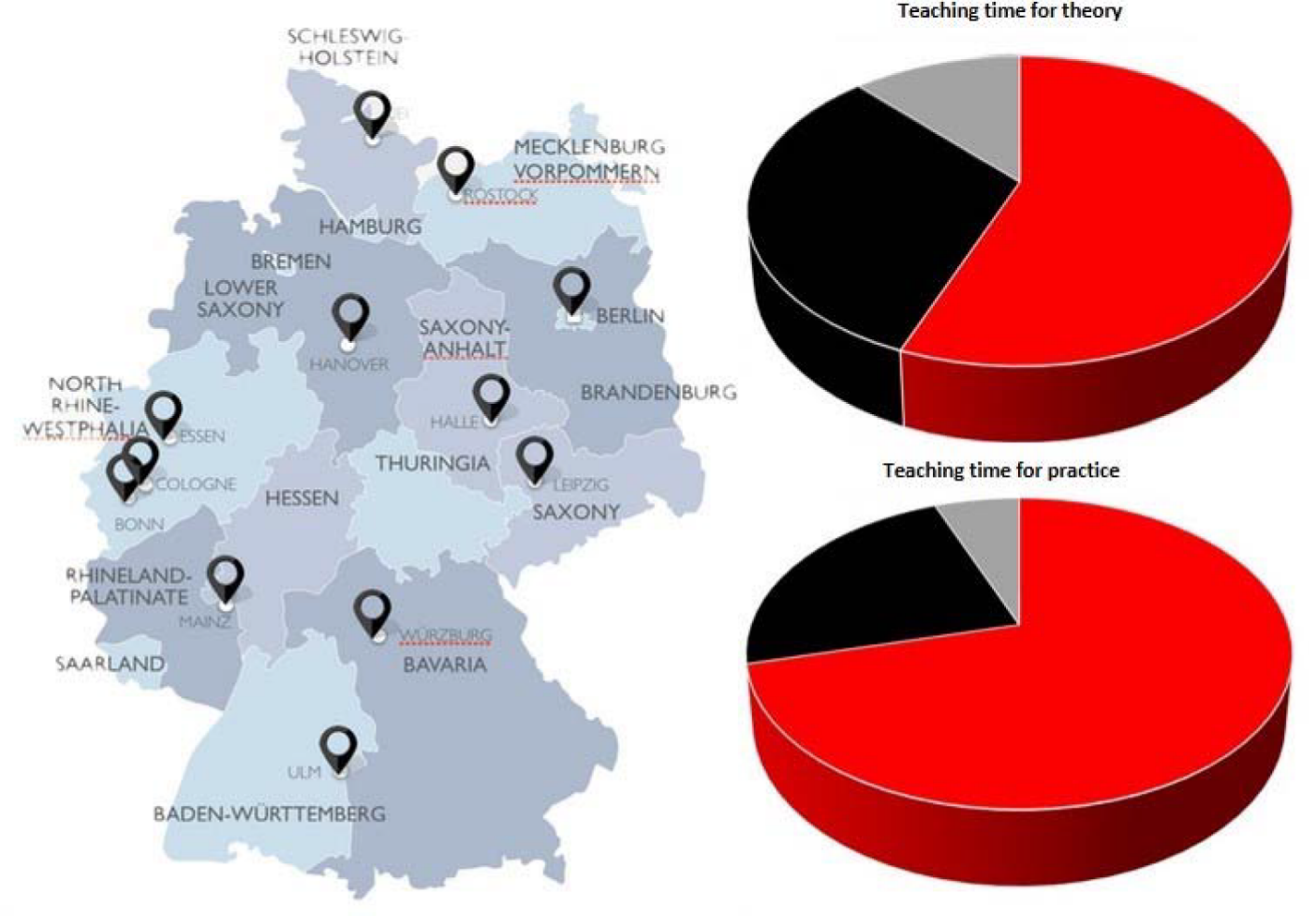
Distribution of teaching time on theory (top right) and practice (bottom right) in actual curricula from eleven medical faculties all over Germany (see flags on map) at the time of our study. Summarised data for clinical-practical (red), clinical-theoretical (black) and socio-economic (grey) disciplines of the clinical part of medical studies are shown.

Both in our survey (see Figure 1 (Fig. 1)) and regarding actual curricula (see Figure 2 (Fig. 2)), the highest amount of teaching time fell upon clinical-practical disciplines. Least teaching time was allocated to socio-economic subjects. The five disciplines with obligatory practical trainings according to the German Medical Licensure Act ÄApprO (i.e. internal medicine, surgery, paediatrics, gynaecology/obstetrics, general medicine) ranked among the top third regarding allocated teaching time for theory or practice (see Figure 1 (Fig. 1)), thereby largely resembling actual curricula. Compared to its representation in actual curricula neurology was rated higher by our study participants (rank 3 and 4 regarding teaching time for theory or practice, respectively). Interestingly, faculties ranking among the top quarter regarding actual state examination results scheduled more teaching time for neurology than the other faculties did. Emergency medicine and anaesthesiology both came off very well regarding the allocation of teaching time for theory or practice (ranks 1 and 8 or 6 and 8, respectively). In the analysed actual curricula, emergency medicine was on the 75%-quartile regarding the amount of practical teaching time. Orthopaedics, dermatology, otorhinolaryngology, urology and ophthalmology were rather mid-level regarding teaching time for theory. However, these disciplines were allocated relatively more time for practical education. 

In general, clinical-practical disciplines were acknowledged more teaching time for practice than for theory. Allocation of teaching time for theory turned out to be quite heterogeneous amongst clinical-theoretical disciplines. Clinical pharmacology/pharmacotherapy and pharmacology/toxicology were acknowledged more generously (ranks 2 and 5), forensics and medical genetics rather parsimoniously (ranks 23 and 24, respectively). However, compared to the pharmacological disciplines, forensics was assigned more time for practical education. Pathology and clinical pathology ranked below the 25%-quartile regarding the allocation of teaching time. In actual curricula, these disciplines were allocated 22% of teaching time for theory and 27% for practice regarding all clinical-theoretical disciplines.

Students’ allocation of rather less teaching time to socio-economic disciplines was in good agreement with actual curricula. Among the socio-economic disciplines, about one quarter of the teaching time for theory and 30% of teaching time for practical education was actually spent on epidemiology/biometrics/informatics. 

#### Motivation for studying and perceived usefulness

**Figure 3:**
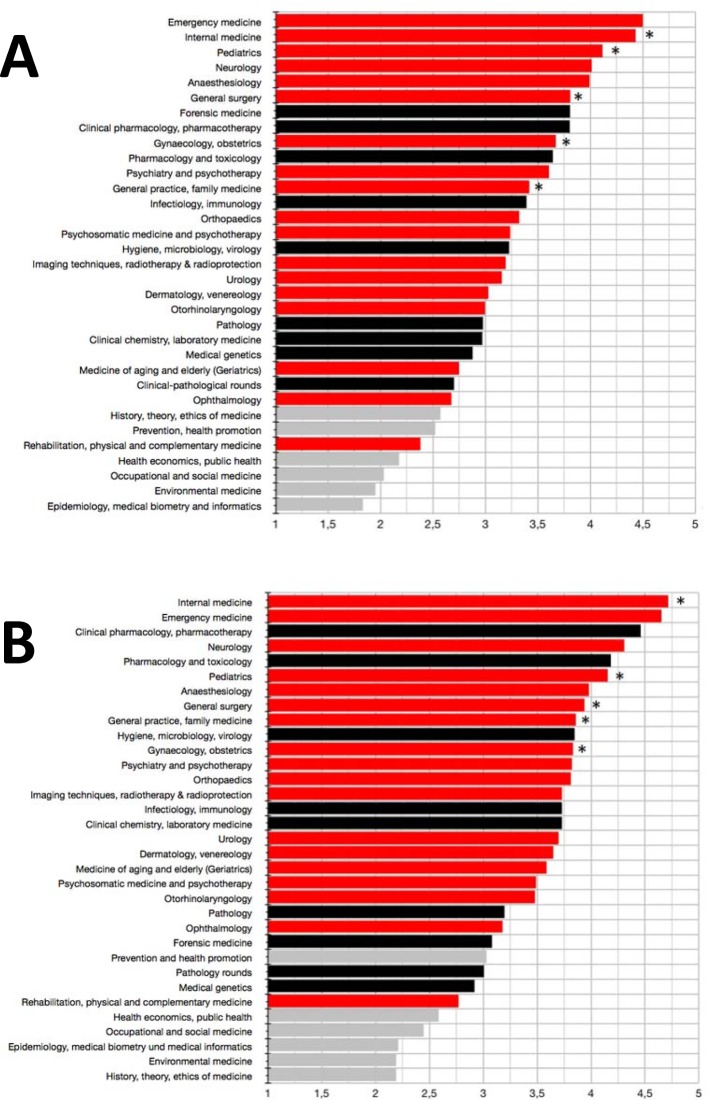
Assessment of motivation for studying (A) and usefulness (B) of clinical-practical (red), clinical-theoretical (black) and socio-economic (grey) disciplines in the clinical part of medical studies. Rating was done via Likert scales (from 1=not interesting to 4=very interesting or 1=not useful at all until 5=very useful, respectively). Mean values are shown. *: disciplines with obligatory practical trainings according to the German Medical Licensure Act (ÄApprO).

Regarding the individual motivation for studying a discipline and its perceived usefulness for daily medical practice, clinical-practical disciplines ranked significantly higher than clinical-theoretical disciplines (see Figure 3 (Fig. 3)). This was particularly true for internal medicine (ranks 2 and 1, respectively). All of the five disciplines with obligatory practical trainings according to ÄApprO ranked amongst the top third regarding usefulness for daily medical practice. Regarding motivation for studying, only general medicine tightly missed the top third (rank 12 of 32). Concerning both motivation and usefulness, emergency medicine, neurology and anaesthesiology performed very well (ranks 1, 4 and 5, and 2, 4 and 7, respectively).

Amongst clinical-theoretical disciplines, clinical pharmacology/pharmacotherapy and pharmacology/toxicology were assessed very positively (ranks 3 and 5). Regarding the motivation to study these disciplines, they ranked amongst the top 10, as the disciplines with obligatory practical trainings did. The assessment of usefulness of hygiene/microbiology/virology is similar to that of general medicine (ranks 10 and 9, respectively). The assessment of motivation for studying forensics was alike that of surgery (ranks 7 and 6, respectively).

#### Perceived preparation for state examination and daily medical routine

**Figure 4:**
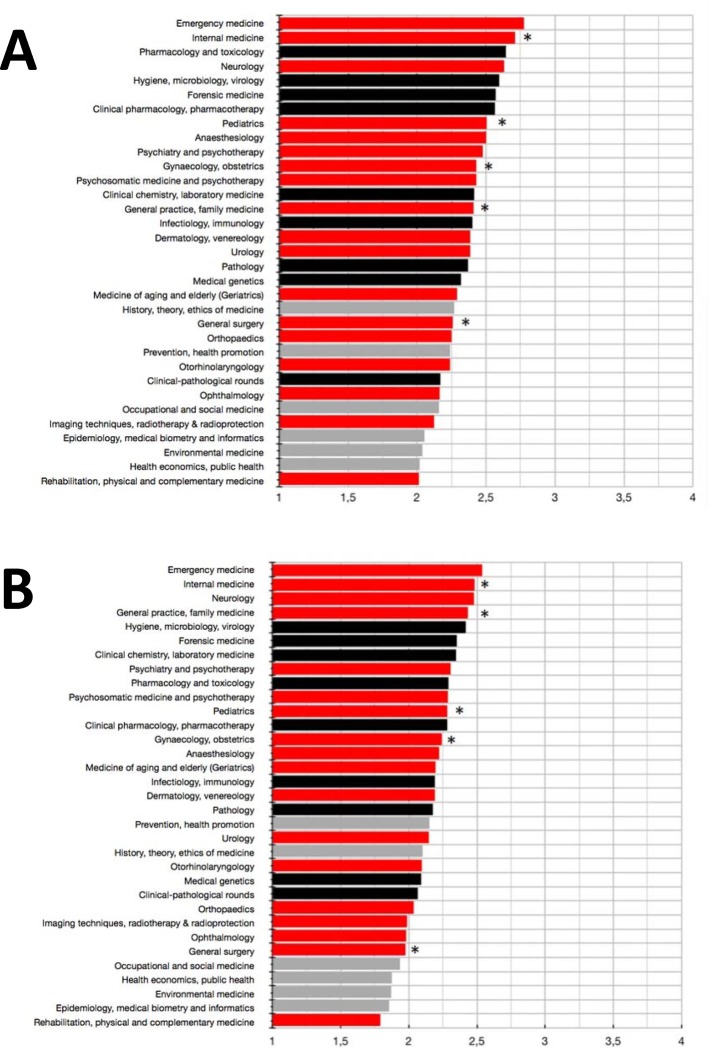
Students’ answers to the questions whether they felt well pepared for state examination (A) or daily medical routine (B) in clinical-practical (red), clinical-theoretical (black) and socio-economic (grey) disciplines of the clinical part of medical studies. Rating was done via Likert scales (from 1=disagree to 4=fully agree). Mean values are shown. *: disciplines with obligatory practical trainings according to the German Medical Licensure Act (ÄApprO).

The participants felt best prepared for state examination and daily medical routine regarding emergency medicine (see Figure 4 (Fig. 4)). Rating of disciplines with obligatory practical trainings was quite heterogeneous. Internal medicine ranked 2nd regarding both, preparation for state examination and daily medical routine, while surgery only ranked 22nd and 28th, respectively. In general medicine, the participants felt better prepared for daily routine than for state examination (ranks 4 and 14, respectively), while regarding paediatrics this was rather opposite (ranks 11 and 8, respectively). Pharmacology/toxicology, hygiene/microbiology/virology, forensics and clinical pharmacology/pharmacotherapy ranked amongst the top 10 regarding perceived examination preparation (ranks 3 and 5-7, respectively). With the exception of clinical pharmacology/pharmacotherapy, these ratings fit the perceived preparation for daily medical routine. Regarding this latter issue clinical chemistry/laboratory medicine was amongst the top 10, too.

When comparing clinical-theoretical and clinical-practical disciplines, we observed no substantial differences regarding the assessment of preparation for state examination or daily medical routine, respectively. However, one has to note that overall, the absolute differences were quite small here. 

#### Relations between items

There was a consistent positive correlation between all survey items (r>0.5). A rather weak correlation was found for allocation of teaching time for practice and preparation for daily medical routine or state examination (r=0.53 and r=0.57, respectively). A rather strong correlation was seen regarding the preparation for daily medical routine and for state examination (r=0.93). In general, students assigned more teaching time to disciplines that they deemed more useful or interesting. On the other hand, the perceived preparation for state examination was rather associated with the motivation for studying a discipline than with its perceived usefulness. 

#### Influence of students’ own future perspectives

**Figure 5:**
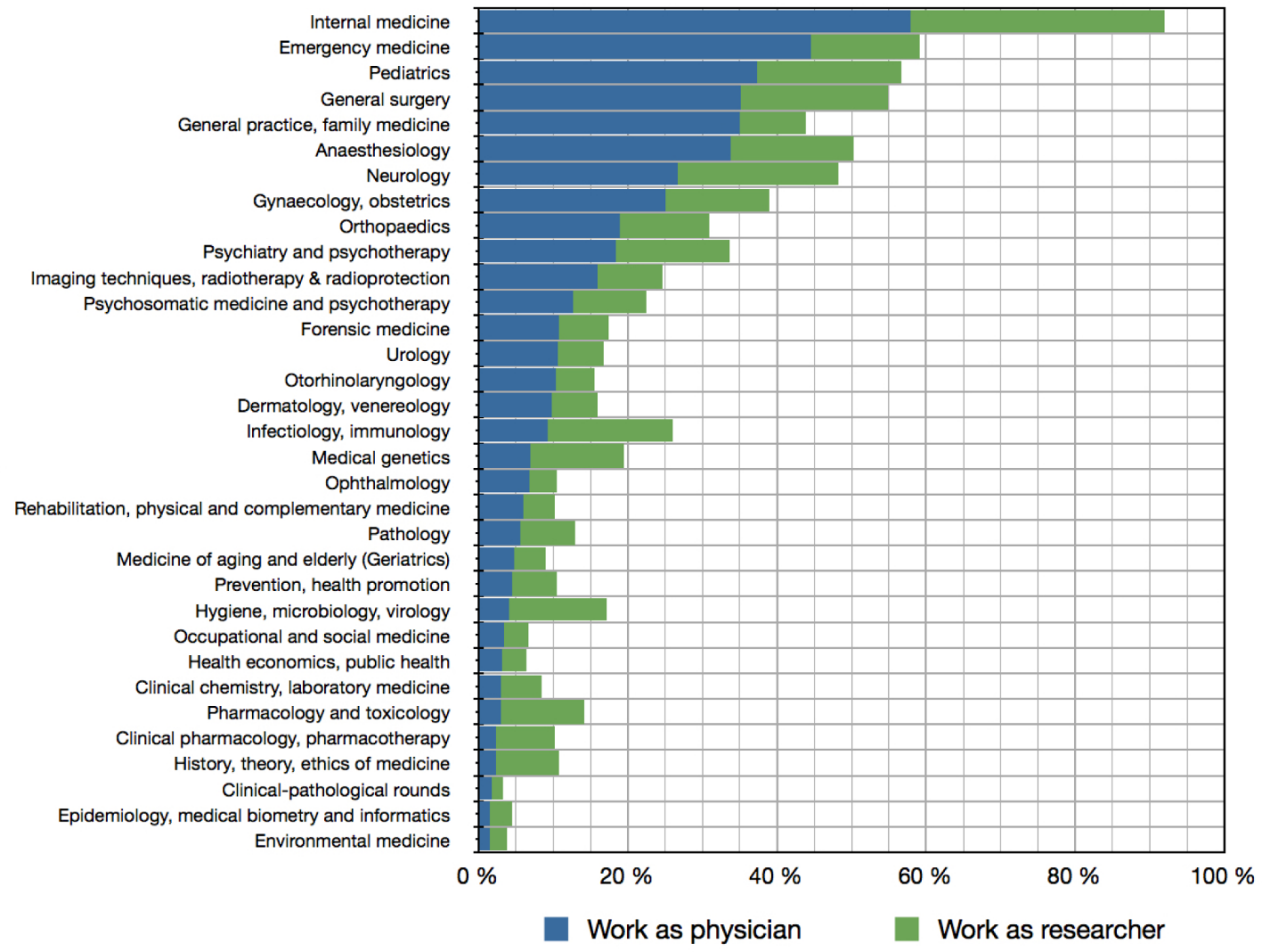
Answers to the questions whether students could imagine a particular discipline for future medical practice (blue) or research (green). Multiple choices were possible.

Neurology, emergency medicine, anaesthesiology, orthopaedics and psychiatry ranked amongst the top 10 disciplines that students could imagine for their future work as a physician (see Figure 5 (Fig. 5)). Fairly the same disciplines were also fancied regarding a future scientific work. Here some clinical-theoretical disciplines were chosen quite high as well (e.g. infectiology/immunology, medical genetics, hygiene/microbiology/virology or pharmacology/toxicology).

**Figure 6:**
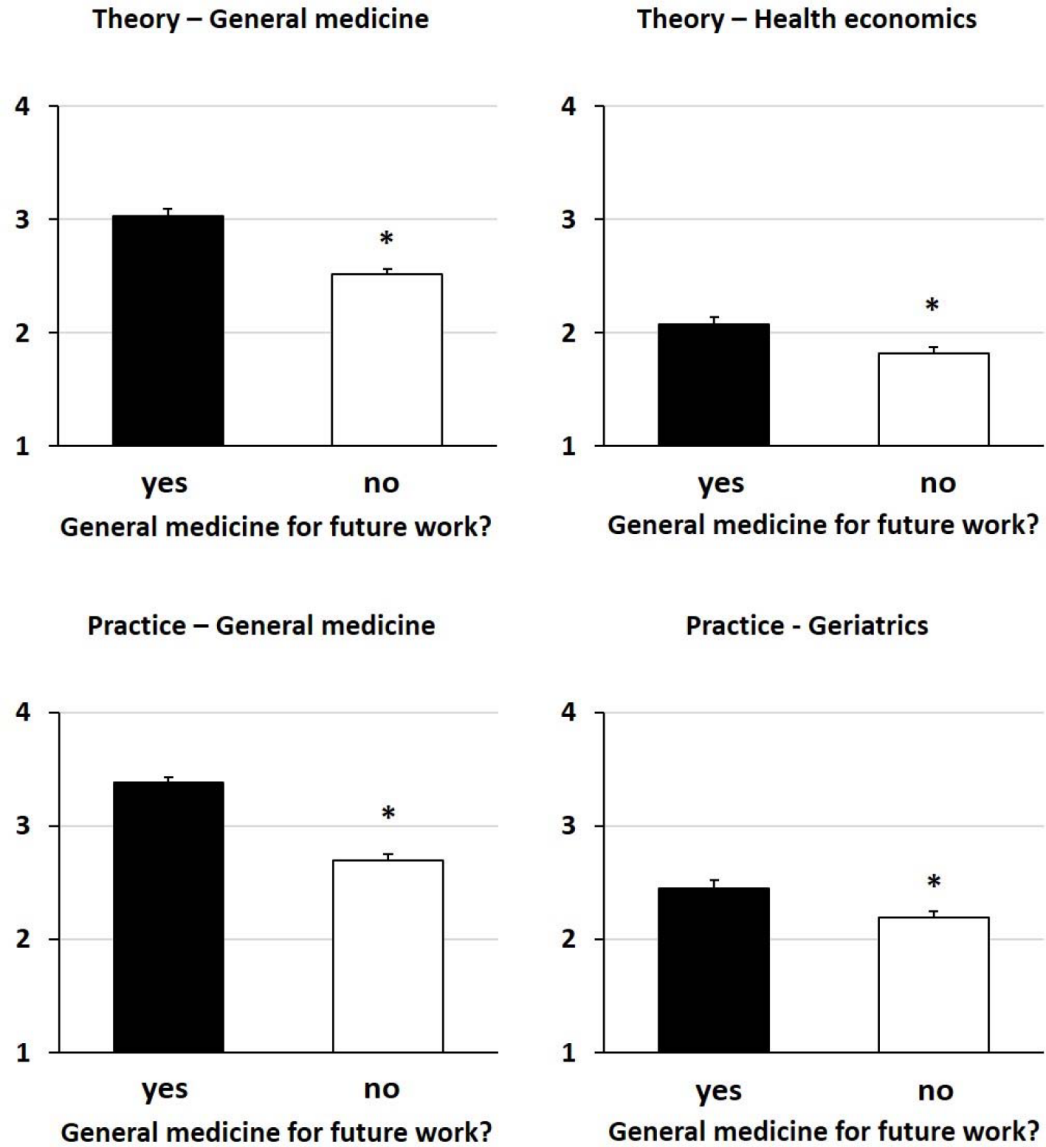
Examples of differences in the allocation of teaching time (from 1=very little to 4=very much) depending on whether students could imagine general medicine for future work as a physician or not. Differences were statistically significant (*) and argue for the plausibility of our data. Mean values and standard errors of the mean are shown.

Students considering internal medicine for future work rated associated, pathophysiologically oriented disciplines significantly higher than other participants regarding usefulness for medical practice (pharmacology/toxicology, hygiene/microbiology/virology) or motivation for studying (clinical chemistry/laboratory medicine, hygiene/microbiology/virology, infectiology/immunology). Students who could imagine surgery for future work rated the usefulness of pathology and clinical pathology higher and assigned more teaching time to these disciplines than others. More than a third (35%) of the participants could imagine general medicine as a future field of practice. Compared to others these students allocated more teaching time to geriatrics, psychosomatics/psychotherapy, health economy or environmental medicine (see Figure 6 (Fig. 6)).

## Discussion

Our survey allows for a comparison of clinical disciplines regarding their assessment by medical students as those who are directly concerned during their studies. In the following, we will first comment on the plausibility of our data, and then we will exemplarily discuss some aspects of potential use for curriculum development.

### Plausibility of results 

The plausibility of our results is exemplarily indicated by the good agreement of teaching time allocation with the assessment of usefulness and motivation for studying (emergency medicine and anaesthesiology) or with the assessment of usefulness for daily medical routine (clinical pharmacology/pharmacotherapy and pharmacology/toxicology), respectively. Of note, the motivation for studying forensics or surgery was similar (ranks 7 and 6, respectively), while regarding the allocation of teaching time forensics fell clearly behind (ranks 23 and 7 for teaching theory, ranks 17 and 2 for practical teaching). Given the rather restrained assessment of usefulness of forensics (rank 24), we suppose some kind of “CSI effect” here (i.e. study motivation is triggered by fascination, not by the anticipated occupational profile) [9].

The future perspectives of the participants seem to be quite realistic: the ten disciplines chosen most often regarding possible future work as a physician are in perfect agreement with the disciplines most physicians in Germany are actually working in [http://www.bundesaerztekammer.de/ueber-uns/aerztestatistik/aerztestatistik-2014/]. Thus, one might assume that the participants also have a quite specific idea of what they should learn to be prepared for their future profession. This is further supported by our finding that students who consider internal medicine for future work have particularly valued associated disciplines, e.g. pharmacology, microbiology, laboratory medicine. Similarly, students fancying general medicine mainly acknowledged the related disciplines geriatrics, psychosomatics/psychotherapy, and health economy.

Given the apparent plausibility of our findings, a cautious interpretation of these data appears to be reasonable.

#### Dissatisfaction with the training?

The rather weak correlation between allocation of time for teaching practice and the assessment of preparation for state examination or daily medical routine suggests that participants were dissatisfied with their practical training in some areas. As an example, regarding surgery the assessment of preparation for daily routine was quite low while students assigned the second most time for teaching practice to this discipline. This seems to reflect the desire to intensify practical training, an interpretation that is supported by a high rating of surgery’s usefulness for medical practice. In contrast, the generous allocation of practical teaching time to emergency medicine rather seems to mean “carry on!”, since here usefulness as well as preparation for state examination and daily medical routine ranked high. Interestingly, pharmacological disciplines performed very well regarding usefulness (clinical pharmacology/pharmacotherapy: rank 3; pharmacology/toxicology: rank 5), while the assessment of preparation for daily medical routine fell behind (ranks 12 and 9, respectively). This dissatisfaction with preparation for daily routine did not translate into a desire for more practical training (ranks 18 and 23, respectively). In contrast, studies from the United Kingdom found that participants desired more practical trainings and applied courses [10], [11]. However, in these studies postgraduates with (more) experience in medical routine were surveyed. In our survey, students assigned much teaching time for theory to the pharmacological disciplines (clinical pharmacology/pharmacotherapy: rank 2; pharmacology/toxicology: rank 4), although they were obviously quite satisfied with their preparation for state examination in these disciplines. Perhaps they assumed that (more) theory may also lead to a better preparation for medical routine. On the other hand, it is quite likely that there was a lack of (experience with) ideas of how to realise applied and practical teaching in pharmacology. However, even German students immediately before starting their final practical year suggested considering practical trainings or ward rounds as teaching formats for (clinical) pharmacology [12].

Of note, the high correlation between the assessment of preparation for state examination and preparation for daily medical routine argues against the interpretation that the overall weak correlation between allocated practical teaching time and the assessment of preparation for state examination is due to a perceived irrelevance of the state examination for actual daily practice. However, one may argue that the predominant (and in many disciplines nearly exclusive) assessment of cognitive knowledge in the state examination often does not give rise to the desire for more practical training (anymore).

#### State examination as a trigger for teaching and learning?

In the curricula of those faculties ranking amongst the top quarter regarding students’ performance in the final state examination, we found significantly more teaching time actually spent on neurology compared to other faculties. Indeed, there has been a quite high proportion of questions on neurological issues in state examinations (about 10% between 2006 and 2010), which may explain the perceived importance of neurology in our study. Regarding surgery, the assessment of preparation for state examination was rather modest (rank 22). This may explain why participants desired rather much teaching time on theory in this discipline (rank 7). The discipline imaging/radiotherapy/radioprotection was allocated a lot of teaching time on theory as well (rank 9). Since the ranking of usefulness for medical practice was only modest (rank 14), here the perception of a rather poor preparation for state examination (rank 29) might have been the trigger for assigning much teaching time as well.

#### Limitations

The proportion of female students in our study (32%) was not representative for medical students in Germany. Though most of the participants were in the clinical part of their medical studies, there might be a “bias” by preclinical students. The response rates from the medical faculties was quite different and the motivation of the participants is unknown. Other confounders cannot be excluded, e.g. state examination results of the particular study sites, the type of study programmes (e.g. reformed or conventional) or whether, for how long and to what extent students had paid tuition fees. Though in Cologne tuition fees seem to have not affected study progress, paying fees obviously made students more critical regarding their studies [13]. A validation of our results would be desirable, e.g. via focus groups or think-aloud technique. This would be most promising on a faculty level, to examine whether and which of the results can be confirmed or applied locally.

## Conclusions

Our study shows that a rather simple survey can reveal interesting data on students’ perceptions and ideas of medical studies. The data seem to be largely plausible and consistent. Their interpretation is likely prone to speculation. A validation would be desirable, but laborious. Nonetheless, we think that on a faculty level (and beyond) results like these should give rise to a more specific inquiry and to constructive discussions, e.g. in the context of curriculum development and (re-) organisation (”curriculum deliberation“).

## Competing interests

The authors declare that they have no competing interests.
